# At the intersection of trust and mistrust: A qualitative analysis of motivators and barriers to research participation at a safety‐net hospital

**DOI:** 10.1111/hex.13726

**Published:** 2023-03-10

**Authors:** Autumn L. Tamlyn, Maria Tjilos, Nicholas A. Bosch, Katherine Gergen Barnett, Rebecca B. Perkins, Allan Walkey, Sabrina A. Assoumou, Benjamin P. Linas, Mari‐Lynn Drainoni

**Affiliations:** ^1^ Boston Medical Center, Section of Infectious Disease Boston MA USA; ^2^ Boston Medical Center, The Pulmonary Center, Department of Medicine Boston MA USA; ^3^ Boston University Chobanian & Avedisian School of Medicine, Section of Pulmonary, Allergy, Sleep, & Critical Care, Department of Medicine Boston MA USA; ^4^ Boston Medical Center, Department of Family Medicine Boston MA USA; ^5^ Boston University Chobanian & Avedisian School of Medicine, Department of Family Medicine Boston MA USA; ^6^ Harvard Center for Primary Care, Center for Primary Care Boston MA USA; ^7^ Aspen Health Innovation Washington DC USA; ^8^ Boston Medical Center, Department of Obstetrics and Gynecology Boston MA USA; ^9^ Boston University Chobanian & Avedisian School of Medicine, Department of Obstetrics and Gynecology Boston MA USA; ^10^ Boston University School of Public Health, Department of Health Law Policy & Management Boston MA USA; ^11^ Boston University Chobanian & Avedisian School of Medicine, Section of Infectious Disease Department of Medicine Boston MA USA; ^12^ Boston University School of Public Health, Department of Epidemiology Boston MA USA

**Keywords:** barriers, mistrust, qualitative, research participation, safety‐net patients, trust

## Abstract

**Introduction:**

The underrepresentation of Black, Indigenous, and People of Color (BIPOC) individuals in healthcare research limits generalizability and contributes to healthcare inequities. Existing barriers and attitudes toward research participation must be addressed to increase the representation of safety net and other underserved populations.

**Methods:**

We conducted semi‐structured qualitative interviews with patients at an urban safety net hospital, focusing on facilitators, barriers, motivators, and preferences for research participation. We conducted direct content analysis guided by an implementation framework and used rapid analysis methods to generate final themes.

**Results:**

We completed 38 interviews and identified six major themes related to preferences for engagement in research participation: (1) wide variation in research recruitment preferences; (2) logistical complexity negatively impacts willingness to participate; (3) risk contributes to hesitation toward research participation; (4) personal/community benefit, interest in study topic, and compensation serve as motivators for research participation; (5) continued participation despite reported shortcomings of informed consent process; and (6) mistrust could be overcome by relationship or credibility of information sources.

**Conclusion:**

Despite barriers to participation in research studies among safety‐net populations, there are also facilitators that can be implemented to increase knowledge and comprehension, ease of participation, and willingness to join research studies. Study teams should vary recruitment and participation methods to ensure equal access to research opportunities.

**Patient/Public Contribution:**

Our analysis methods and study progress were presented to individuals within the Boston Medical Center healthcare system. Through this process community engagement specialists, clinical experts, research directors, and others with significant experience working with safety‐net populations supported data interpretation and provided recommendations for action following the dissemination of data.

## 1 INTRODUCTION

Structural racism faced by Black, Indigenous, and People of Color (BIPOC) is present in multiple arenas, and the medical field is no exception.^1^, ^2^, ^3^, ^4^ In 1993, the National Institutes of Health acknowledged the need to increase diversity in healthcare research when they mandated the inclusion of women and marginalized groups in NIH‐funded clinical trials.^5^ However, there is a clear lack of progress in the implementation of this policy as nearly 20 years later, BIPOC individuals are still underrepresented in healthcare‐related research studies.^6^, ^7^, ^8^ For example, of more than 300,000 enrollees in trials submitted to the US Food and Drug Administration between 2008 and 2017, a mere 4% represented the Black population.^9^

Clinical studies that exclude large segments of the population are not generalizable and do not lead to a knowledge base that is inclusive of the entire population. Without inclusivity, the medical system cannot deliver on the promise of tailored medicine or targeted therapies. In addition, within this infrastructure, underrepresented communities do not share equally in the benefits of research which in turn exacerbates health inequities.^10^, ^11^, ^12^, ^13^, ^14^, ^15^ This issue became clear early in the coronavirus disease 2019 (COVID‐19) pandemic when US death rates among Black and Latino individuals were threefold higher compared with White individuals.^16^ This trend is exhibited more chronically in diseases such as breast cancer, where African American women have higher mortality rates than their White counterparts despite having lower incidence.^17^ The consequences of underrepresentation call for immediate action and an increased focus on the inclusion of BIPOC individuals in healthcare research.^18^, ^19^, ^20^, ^21^, ^22^

While the need for better inclusion of BIPOC populations in healthcare research is clear, a myriad of barriers to achieving sufficient representation exist and lead to challenges with the recruitment and retention of samples that are adequate for making race‐stratified conclusions.^6^, ^15^, ^23^, ^24^, ^25^, ^26^ Barriers include, but are not limited to, language/literacy, socioeconomic status, the complexity of informed consent, and limited access to technology.^23^, ^25^, ^27^, ^28^, ^29^, ^30^ Further, systems of oppression and historical maltreatment of minoritized communities have resulted in a culture of mistrust in scientific institutions that contributes to hesitancy toward participating in healthcare research.^8^, ^28^, ^31^

Safety‐net hospitals and clinics are facilities that primarily serve patients from traditionally marginalized communities, including individuals of lower socioeconomic status, who are uninsured, who receive Medicaid benefits, and who identify as BIPOC individuals.^23^, ^32^ This patient population makes safety‐net facilities a potentially valuable venue for future clinical research. However, the United States does not have a long track record of conducting rigorous, high‐quality clinical trials in safety‐net venues. Rather, sponsors conduct most studies in large, academic medical centres with low proportions of publicly insured and BIPOC populations.^33^ Through qualitative analysis of previous experiences, the current study aims to identify facilitators, barriers, motivators, and preferences for research participation among patients who receive their medical care in a safety‐net setting. Knowledge gained from this research can help to support the creation of approaches to increase the representation of safety‐net populations in research studies.

## 2 METHODS

### 2.1 Study setting and data collection

We conducted semi‐structured qualitative interviews with patients at Boston Medical Center (BMC), focusing on facilitators, barriers, motivators, and preferences for research participation. BMC is the largest safety‐net hospital in New England and the Accountable Care Organization (ACO) for Massachusetts Medicaid in Eastern Massachusetts.^34^ Nearly 75% of BMC patients identify as medically underserved, and approximately 65% utilize public insurance.^34^, ^35^ The BMC patient population reflects the racial and ethnic diversity of Boston, where approximately 30% of patients do not speak English as a primary language, about 30% identify as Black, and about a quarter identify as Latino.^34^, ^35^, ^36^ Additionally, 7% of patients at BMC experience unstable housing or homelessness.^35^

As an academic medical centre focused on caring for a safety‐net population, we know that research is important to help us provide high‐quality, equitable, and evidence‐based care to our patients. Many of our faculty conduct research with our patient population.^37^, ^38^, ^39^ Additionally, BMC has an active clinical trials network office that focuses on conducting studies with our patients. Research opportunities are made available to patients through a variety of platforms including direct referral/conversations during clinical visits, flyers posted in clinic exam rooms and waiting rooms, and opportunities presented on the website.

We created and used a semi‐structured interview guide to gain better insight into participants' previous experiences with research, understanding of the research process, and experiences with informed consent. We also encouraged participants to reflect on the process of learning about research studies, preferences for future research, and factors that influenced the decision to, or not to, participate in a research study.

We recruited participants from a pool of 74 individuals who had previously either participated in a randomized control trial (APPEX) or qualitative interviews assessing experiences with the hospital discharge process (CIIS Equity) at BMC and had agreed to be contacted for future research opportunities.^37^, ^40^ Of the 74 eligible individuals, 74% (*n* = 55) were able to be contacted, and 60% (*n* = 44) agreed to participate in the current study. For those willing to participate, we scheduled an interview time and sent informed consent information via email or mail.

We conducted interviews with 38 individuals by telephone in either English or Spanish. We recorded all interviews and documented verbal consent at the time of the interview. We provided participants with a $50 gift card as compensation for their participation. The Boston Medical Center and Boston University Medical Campus Institutional Review Board approved all study procedures.

### 2.2 Data analysis

A professional transcription company transcribed verbatim all audio recordings; interviews completed in Spanish were then translated into English for analysis. After transcription, we reviewed all transcripts against the audio recordings for clarity and accuracy.

We conducted a directed content analysis guided by the Consolidation Framework for Implementation Research (CFIR).^41^ CFIR is a comprehensive framework that incorporates 39 constructs across five broad domains thought to influence implementation success: [1] intervention characteristics, [2] inner setting, [3] outer setting, [4] characteristics of the individuals, and [5] implementation process.^41^ CFIR constructs were identified as a priori codes for an initial codebook. Before beginning coding, three research team members (A. T., M. T., M. D.) reviewed and discussed the CFIR constructs and coding definitions to reach a collective understanding of the codes. Two study team members (A. T. and M. T.) then independently coded an initial set of transcripts, making modifications to the codebook as needed to capture the nuances of research participation. The two coders met approximately bi‐weekly to review coding and reach a consensus. Once consensus was reached, the two coders independently coded the remaining transcripts, meeting if discrepancies arose. We completed all coding in Nvivo 12.7.0 (2019).

Once coding was complete, we used rapid qualitative data analysis methods to produce expedient and actionable results.^42^, ^43^, ^44^ To complete rapid analysis, we followed the following steps: created analysis summaries for each transcript; rated analysis summaries with positive/negative and weak/strong ratings to determine the influence of each summary on participation behaviours; aggregated summary analyses in each thematic area; and generated final themes using the aggregated summaries. At each step of the rapid analysis process, we selected illustrative quotes to ensure conceptual clarity was maintained. Consistent with the initial coding, two study team members (A. T. and M. T.) worked together for each phase of rapid analysis until consensus was reached, at which point the remaining work was divided and completed independently. Two study team members (A. T. and M. T.) completed the generation of final themes individually and then compared and discussed with the senior author to produce final themes. In the results section, we report all transcribed dialogue verbatim and set direct quotes apart using quotation marks. We differentiate quotes of individuals recruited from APPEX or CIIS Equity studies with ‘A’ and ‘E’ respectively, combined with the participant ID number.

## 3 RESULTS

### 3.1 Characteristics of the study population

A total of 38 individuals participated in the current study; of these, 36 were interviewed in English and two in Spanish. Among study participants, 53% identified as Black/African American and 29% identified as White. Just under 25% additionally identified as Latino. The mean age of participants was 51.6 (SD 18.4) years, with a range of 18–83. Just over 60% of the sample was female (Table 1).

**Table 1:** Demographics of participants (*N* = 38).

	*N*	%
Sex		
Male	15	39.5
Female	23	60.5
Race		
Black/African American	20	52.6
White	11	28.9
Other/unknown	7	18.4
Hispanic/Latino	8	21.1
Mean age, years (SD)	51.6 (18.4)	

### 3.2 Overview of themes

**Figure 1:**
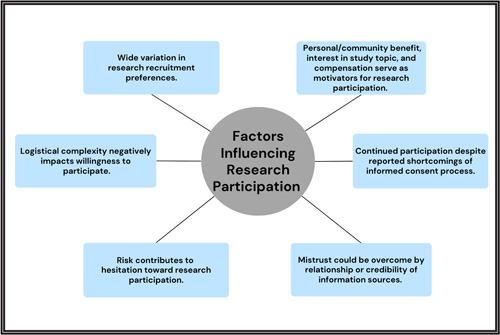
Factors influencing research participation: Overview of themes.

We identified six major themes regarding research participation (Figure 1).

#### 3.2.1 Theme 1: Wide variation in research recruitment preferences

Participants indicated wide variation in how they would prefer to be recruited for research studies. Many preferred face‐to‐face or telephone recruitment methods due to stated ease of communication: ‘I'd prefer a phone call. That way I would feel like I can hear the words, you know? I mean, they can explain, you can talk to each other, you know?… I prefer it by phone’ (E#25). One participant who preferred in‐person recruitment said: ‘… the in‐person one where they were visiting hospital, I think it's kind of effective because it's something that is –and I'm going through in the moment and I will have a fresh experience’ (E#06).

While a few participants reported a preference for recruitment via flyers and advertisements, these methods were typically believed to be easily overlooked: ‘We see flyers in the street and everything, and we just don't pay attention to them’ (A#28). Despite online recruitment being viewed as convenient and wide‐reaching, it also brought up issues related to the perceived value of the research study. When asked if online recruitment was effective, one participant stated, ‘I'd say yes because everybody's on social media for the most part and then I'd say no because well, from the ones that I've seen on social media, they're not really informative or they don't really make any impact, any change’ (E#29). Another participant stated, ‘I feel like posting anything on social media makes people think it is not serious’ (E#36).

#### 3.2.2 Theme 2: Logistical complexity negatively impacts willingness to participate

Convenience and logistics of participation influenced participants' decision to participate in research. When asked why they did or did not elect to participate, one participant said, ‘I think that one of the most important things is like the convenience of it’ (E#06). Included in concerns about complexity were the number of study sessions, distance to the study site, study duration, and intricacy of research activities. For example, ‘Well, if it's really involved, going to take an hour and get to do it again another hour, another week, another hour. If it's too involved, I think you know that is a long time to sit down’ (E#05) In contrast, simple studies of short duration were viewed much more favourably, ‘…a quick survey I wouldn't mind doing. I feel like if anything longer, I was like, oh, I don't want to do that’ (A#25).

Some participants reported that remote participation increased convenience and therefore would have a positive impact on willingness to participate, ‘if it's something online, then I would definitely do it’ (A#06). Remote participation via phone was also viewed favourably by some participants: ‘like a phone call that is convenient to do, I can do that from wherever I am’ (E#36). Additionally, remote participation eliminated barriers related to travel and transportation, ‘Well, it all depends on where I would have to go and how I would get there and get back home. If I had to go somewhere to do the research, that would be a big part of it. You'd have to make sure I get transportation there and back home’ (E#15). While remote participation was reported to be logistically easier, some forms of remote participation were preferred. For example, some participants preferred studies conducted via telephone as opposed to online due to the complexity of technology, ‘No, phone is alright because I'm not that savvy in technology. So phone is easier for me’ (E#09). Other participants reported hesitation for remote participation due to language barriers or because they were more trusting of in‐person interaction. When asked about their preferred method of participation, one participant stated, ‘in‐person, I feel more comfortable with the people’ (A#30).

#### 3.2.3 Theme 3: Risk contributes to hesitation toward participation in research studies

Participants expressed increased hesitation to participate in studies that they perceived as higher risk. Medical studies requiring a blood draw or other tests were viewed as having relatively minimal risk: ‘There's very little risk of doing a pap smear, or blood draw. I mean, there's always some off the wall, you might get an infection’ (A#15). In contrast, studies during which a participant would be subject to taking medication or other types of treatment were viewed as having higher risk, and therefore participants reported greater hesitation, ‘It just seems dangerous and I don't want to have to, you know, put myself in any unnecessary danger when it comes to experimental drugs’ (E#27). Another participant reported dropping out of a research study due to concerns of potential risk involved with repeated testing, ‘Well, I wasn't sure how it would affect me, because that was the first time I've ever had one. I'm not sure what continuous MRIs would do to me internally’ (E#09). For some individuals, having to take medication was reported to end their interest in participating. In learning about a study one participant reported, ‘Once they said I had to take pills, I didn't want to do it’ (A#22).

#### 3.2.4 Theme 4: Personal/community benefit, interest in the study topic, and compensation serve as motivators for research participation

Several motivators were cited by participants, including personal and/or community benefit, interest in the study topic, and compensation. Participants expressed a desire to contribute to research that serves the greater good, ‘…I just want to get involved in the community so by taking on that research, it's my contribution to the societies’ (E#01). Participants also reported being more likely to participate in studies of personal relevance or that they perceived to be more interesting, ‘…the importance of whatever it is they're researching as it applies to my community and my life makes that more interesting to me to participate’ (E#19). Participants additionally reported that interest in a study topic has potential to overcome other barriers or preferences. For example, one participant who typically preferred remote research opportunities stated, ‘if I have to go like [to a] specific location, it actually depends on that one. It depends if I really, really, really like [what] the research is about, then I will consider doing it’ (A#06).

Almost all participants described compensation as an important motivator, ‘Honestly, it's because of money. I don't work right now because I just had a knee surgery…. I was short of money’ (E#21). Even those who stated that compensation was less important still noted that it was appreciated or they could understand why it would be an important factor for other participants, ‘I think it kind of helps, but no, it's not my, you know, it's not my main motivation’ (A#24).

#### 3.2.5 Theme 5: Continued participation despite reported shortcomings of the informed consent process

The informed consent process was often reported as complex and difficult to understand. One participant said, ‘I think I understood only 40%. I really want to understand everything’ (A#06). For those who did recall elements of the informed consent process, it was often reported with little recollection of detail, such as, ‘All I remember was the hospital asking me if I wanted to try the same drugs that President Trump had been given. And I said, yes’ (A#19). When asked if they recalled the informed consent process one participant responded, ‘I don't clearly remember all that they told me. I just know that it was helping the advance of Alzheimer's and dementia. Things like that’ (E#09). Furthermore, being ill was a barrier to understanding informed consent, ‘I was really sick, so I was kinda just like, yeah, sure’ (A#32). Despite this lack of understanding or recollection to detail, participants often still report continuing with their participation in the research study, ‘I remember that like, it wouldn't help me but it could help people further down the road. And – I'm trying to remember. It was—you know, it was—that's all I kinda remember. So I said yeah of course I would help out’ (A#32).

#### 3.2.6 Theme 6: Mistrust could be overcome by the relationship with or credibility of information sources

Many participants described a lack of trust in both research and the broader healthcare system. Participants cited historical research studies, previous maltreatment of BIPOC communities, or referenced previous negative personal experiences with healthcare or research. For example, ‘I read somewhere in the past there was something going on in the Black community where they were doing research for syphilis…So the effect, I had that in my mind you know, I don't waste my time with the research’ (E#01). One participant reported a previous negative experience of participating in research, ‘So, he ended up giving me like 20 something needles sticks because he couldn't get my blood…And because of that, I had dropped out [of] the study not even knowing that I had participated in a study’ (A#19). References such as these were often told with significant emotion and explained to be a clear cause of hesitancy toward future participation.

Despite existing hesitancy participants indicated that the quality of interaction with staff during recruitment impacted their willingness to participate. Whereas positive interactions increased the likelihood of participation, negative experiences contributed to decreased enthusiasm for participating, ‘It's like she looked down on me, like she was treating somebody lesser than her. Hey, you're in the medical profession, you're supposed to treat people equally… regardless of where they come from, their background, their skin color or anything’ (E#05). However, in the setting of positive interactions, participants often reported they were willing to participate because researchers or study staff were perceived as friendly and/or welcoming, ‘I remember they were really cool…the way they were speaking to me, and they were constantly checking up on me to see if I needed anything. They were just really friendly people’ (A#12).

In addition to positive interaction, personal familiarity with healthcare staff and trusted healthcare institutions increased comfortability in participation, ‘I didn't have a problem with it, because me and my doctor are very close’ (E#16). Being informed of research studies by credible sources, or by individuals or institutions with which the potential participant has a longstanding relationship, was indicated to lead to trust in the research process and motivate participation. When asked why they participated in the current study, one participant stated ‘…because number one, I like Boston Medical Center…’ (E#31). Lastly, participants reported that when clinicians were involved in the research process, it left the impression that the research was positive and for the benefit of others, ‘If it was doctors… I think that it was gonna be ideally beneficial or be used for like good purposes’ (E#06).

## 4 DISCUSSION

We employed qualitative methods and the CIFR framework to identify factors that influenced decisions to participate in research studies from the perspective of diverse safety‐net hospital patients. One notable finding is that the concerns about research among patients in a safety‐net setting are similar to those of research participants more generally.^45^, ^46^ Our participants reported barriers to participation, such as securing transportation to a study site and commitment to a study of long duration. However, participants indicated that providing financial compensation or other support for participation, such as reimbursement for transportation, made participation easier and increased their willingness to participate. Participants indicated additional facilitators to include recruitment in familiar settings, in‐person recruitment, and increasing factors of convenience, such as allowing for remote participation. While catering to individual preferences and creating opportunities for high‐quality interaction was found to increase the likelihood of participation, the current findings highlight that a critical ingredient for study recruitment and retention is trust. Lastly, in regard to obtaining informed consent, participants reported this process to be challenging and recalled little detail, feeling that upon agreement for participation, they had less than a full understanding of study details.

Our findings are consistent with previous research which found that minority populations experience increased challenges that limit their ability to participate in research studies.^27^ Our results are also consistent with reported facilitators that improve willingness to participate, such as utilizing familiar settings, in‐person recruitment, and capitalizing on convenience, such as with remote participation.^15^, ^26^, ^28^, ^47^ While these facilitators have been shown to increase their willingness to engage with research, our findings point to the limits of using a one‐size‐fits‐all model when recruiting in the safety‐net setting. The wide variation of recruitment preferences shows that what may act as a facilitator for a specific subset of the safety‐net population may not be the same for another. For example, while some participants reported a preference for remote participation due to scheduling flexibility and ease of participation, others reported that remote participation created additional barriers due to perceived complexity, limited access to technology, language barriers, or lack of human interaction. This finding indicates that researchers should utilize varied recruitment and participation methods to ensure equal access to research opportunities for safety‐net populations.

Our findings show that the mechanics of participating in research studies, such as recruitment, risk, and complexity, are a cause for hesitation among safety‐net participants. As aforementioned, concerns shared by safety‐net patients are also expressed outside of the safety‐net setting.^45^, ^46^ If researchers want to increase participation from diverse communities, then they also need to work on simplifying the logistics of the research process. It may be valuable for researchers to reflect upon what aspects of study participation feel logistically complex for participants, and if there are ways to streamline the participation processes. In the safety‐net setting, implementation of this practice could be designing protocols that are sensitive to the needs of the patient population, such as utilizing home visits or community‐based sites that would make participation possible while simultaneously meeting needs for childcare, work, or other commitments.

Based on our findings, there is a clear need for inclusivity and improvement of informed consent practices that effectively communicate critical study details to participants in a manner that informs their real‐world decision‐making. Current operating procedures for many clinical studies struggle to provide such communication. Study documents developed entirely in English and then translated into other languages, for example, are often not the most effective form of knowledge transfer. Nor are documents framed with procedural language and legal complexities. Study procedures, however, typically require adherence to such documents in structuring the informed consent process. Identifying ways to tailor informed consent discussions to participant needs and queries without compromising on the quality of information being given is critical for making progress in increasing the diversity of research study participation.

As it is known that trust in the patient‐provider relationship and one's healthcare organization is critical when making healthcare decisions, existing medical mistrust must be considered when conducting research.^29^, ^48^, ^49^ Our findings show that trustworthiness can be built during the research process through positive experience, communication, and connection, or can be pre‐existing through a relationship with an individual, sector, or institution. Knowing the positive impact that trustworthiness can have on an individual's decision to participate in research, methods to increase trustworthiness should be considered and utilized to support increasing representation of safety‐net populations in healthcare research. To aid in this endeavour, improving the services provided at all levels will support patients in feeling cared for and allow for trustworthiness to be built by hospitals and providers in general. If patients believe their clinicians and their hospital provide them with the best possible care, they will be more likely to believe that research is legitimate and beneficial to themselves and their communities. In turn, the research enterprise will have a stronger foundation to stand on when recruiting for research studies. Future research should investigate the connection between research opportunities being presented in practice and the impact on participation interest.

Based on our findings, we suggest that a foot‐in‐the‐door (FITD) approach could yield positive results for achieving increased participation of safety‐net populations in healthcare research. FITD is a concept which suggests that engagement in a small commitment may increase the likelihood of engagement in a more substantial commitment later on.^50^, ^51^, ^52^ While this concept has been studied extensively in psychology and other fields, it has not been examined in the context of engaging patients in research.^52^, ^53^, ^54^, ^55^ As the current study found a preference for short and convenient studies as compared to those of more increased complexity or risk, we propose that this willingness to participate in simple studies could be a way of introducing individuals to research, creating the opportunity for familiarity with the research process, and building trustworthiness of scientific institutions with the community. While more research is needed, a FITD approach could utilize aspects of positive experience, education, and trust to eventually increase participants' comfortability in joining more complex studies.

This study has several limitations. First, all recruited participants had previously agreed to participate in at least one research study, and we did not obtain the perspective of those who would not agree to participate in the research. However, utilizing participants who had previously been involved in research studies also supported the validity of our findings as they could provide a perspective that was based on lived experience, rather than assumptions. Second, several participants reported no recollection of previous participation and therefore did not have a context to discuss questions related to previous participation in research, thus there may be recall bias. In addition to the recollection of study participation, contributions regarding the informed consent process may have been impacted by the passing of time, and therefore participant quotes may not reflect their understanding at the time of consent. The fact that only two individuals elected to complete the interview in Spanish was another limitation of our study and may impact the generalizability of our data to Spanish‐speaking populations who do not feel comfortable conversing in English. Finally, our study was conducted at a single institution and may not be generalizable to other safety‐net settings. Despite these limitations, our study provides valuable insight into strategies for recruitment, motivators for participation, and approaches to build trustworthiness in research among safety‐net populations.

## 5 CONCLUSION

While there are many barriers to participation in research studies among safety‐net populations, there are also facilitators that can be implemented to increase knowledge and comprehension, ease of participation, and willingness to join research studies. To improve recruitment of BIPOC and other underrepresented populations into research studies, researchers should ensure that: patients are treated with respect, compensation is adequate for the time and inconvenience involved, informed consent documents are understandable and available in the participants' primary language, and remote participation opportunities (online or phone) are available when appropriate. As safety‐net participants expressed a wide variety of preferences, study teams must avoid using a one‐size‐fits all mentality and may need to vary recruitment and participation methods to ensure equal access to research opportunities.

## AUTHOR CONTRIBUTIONS

**Autumn L. Tamlyn**: Conceptualization; methodology; formal analysis; writing—original draft, review and editing; **Maria Tjilos**: Conceptualization; methodology; formal analysis; writing—review and editing. **Nicholas A. Bosch**: Writing—review and editing. **Katherine Gergen Barnett**: Writing—review and editing; **Rebecca B. Perkins**: Writing—review and editing. **Allan Walkey**: Writing—review and editing. **Sabrina A. Assoumou**: Writing—review and editing. **Benjamin P. Linas**: Conceptualization; writing—review and editing. **Mari‐Lynn Drainoni**: Conceptualization; methodology; formal analysis; writing – original draft; supervision; review and editing.

## Data Availability

The data that support the findings of this study are available upon request from the Institutional Review Board at Boston University Medical Campus and Boston Medical Center at medirb@bu.edu.
